# Ensembl comparative genomics resources

**DOI:** 10.1093/database/baw053

**Published:** 2016-05-02

**Authors:** Javier Herrero, Matthieu Muffato, Kathryn Beal, Stephen Fitzgerald, Leo Gordon, Miguel Pignatelli, Albert J. Vilella, Stephen M. J. Searle, Ridwan Amode, Simon Brent, William Spooner, Eugene Kulesha, Andrew Yates, Paul Flicek

doi:10.1093/database/bav096

**ncRNA orthologies in the vertebrate lineage**

**Albert J. Vilella, Matthieu Muffato, Leo Gordon, Simon White, Paul Flicek and Javier Herrero**

doi:10.1093/database/bav127

These interconnected articles were originally published with incorrect references 45 and 44, respectively. In the manuscript ‘Ensembl comparative genomics resources’, reference 45 to the authors’ related paper should have read as follows:

45. Pignatelli,M., Vilella,A.J., Muffato,M. *et al.* (2016) ncRNA orthologies in the vertebrate lineage. *Database* (Oxford), 2016, bav127.

In the manuscript ‘ncRNA orthologies in the vertebrate lineage’, reference 44 to the authors’ related paper should have read as follows:

44. Herrero,J., Muffato,M., Beal,K. *et al.* (2016) Ensembl comparative genomics resources. *Database* (Oxford), 2016, bav096.

The publisher apologizes for this error.

